# *Scorias spongiosa* Polysaccharides Promote the Antioxidant and Anti-Inflammatory Capacity and Its Effect on Intestinal Microbiota in Mice

**DOI:** 10.3389/fmicb.2022.865396

**Published:** 2022-03-10

**Authors:** Yingyin Xu, Zhiyuan Zhang, Huiyu Feng, Jie Tang, Weihong Peng, Ying Chen, Jie Zhou, Yong Wang

**Affiliations:** ^1^Sichuan Institute of Edible Fungi, Chengdu, China; ^2^National-Local Joint Engineering Laboratory of Breeding and Cultivation of Edible and Medicinal Fungi, Chengdu, China; ^3^Scientific Observing and Experimental Station of Agro-microbial Resource and Utilization in Southwest China, Ministry of Agriculture, Chengdu, China; ^4^College of Food and Biological Engineering, Chengdu University, Chengdu, China

**Keywords:** *Scorias spongiosa* polysaccharides, antioxidant, anti-inflammatory, intestinal microbiota, mice

## Abstract

*Scorias spongiosa*, as an edible fungus, has multiple health benefits. However, the effects of *S. spongiosa* on intestinal health are rarely explored. Hence, our study aims to elaborate on the influences of *S. spongiosa* polysaccharides (SSPs) on antioxidant, anti-inflammatory, and intestinal microflora in C57BL/6J mice. In the present study, 18 male mice were randomly distributed into three groups: (1) Control group (CON); (2) Low dose SSPs group (LSSP); (3) High dose SSPs group (HSSP). After 14-day administration, the jejunum and serum samples were collected for detection. The results showed that SSPs exert no effects on the growth performance of mice regardless of doses. Meanwhile, SSPs administration reduced the serum pro-inflammatory cytokines and elevated the anti-inflammatory cytokines. Moreover, the antioxidant capacity was elevated by SSPs administration, as evidenced by the increased contents of T-AOC, GSH-Px, and the decreased content of MDA. Mechanistically, the administration of SSPs enhanced the protein abundances of p-Nrf2, Keap1, and HO-1 in mice. The results of 16S rDNA demonstrated that the microbial community and composition were altered by SSPs administration. To summarize, SSPs benefit intestinal health in C57BL/6J mice *via* a mechanism that involves elevating antioxidant and anti-inflammatory activities and regulating intestinal microbiota.

## Introduction

The intestinal microbiota is considered to be a dynamic organ that plays an important role in maintaining host health, which includes Firmicutes, Bacteroidetes, Actinobacteria, Fusobacteria, and so on (Cheng et al., 2020; Wu et al., 2022). The microbiota can regulate the proliferation and differentiation of intestinal epithelial cells (IECs) and be conducive to intestinal absorption of digestible nutrients (Lin et al., 2021; Chen et al., 2021a). Moreover, intestinal flora can promote the secretion of SIgA, a very important immunoglobulin in intestinal mucosal immunity, to prevent bacterial adhesion and invasion and to maintain the integrity of the intestinal mucosal layer (Bain and Cerovic, 2020). However, studies have indicated that the disturbance of intestinal microbiota may participate in the process of disease development, such as diabetes mellitus (DM), obesity, inflammatory bowel disease (IBD), cardiovascular disease (CVD), tumor, and mental diseases (Dong et al., 2019; Villeger et al., 2019; Alvarez-Vieites et al., 2020; Suslov et al., 2021). Therefore, finding effective natural bioactive ingredients to keep the balance of intestinal microbiota is of utmost interest.

The edible basidiomycete *Scorias spongiosa*, which belongs to the genus *Scorias Fr.*, (1825), was discovered by He in 2011 and is considered as a new record species after a pure culture experiment and internal transcribed spacer (ITS) sequence analysis (Zhong et al., 2020). *Scorias spongiosa* polysaccharides (SSPs), a chemical bioactive compound, were secreted through various stimulating agents, such as surfactants and organic solvents (Wu et al., 2018). Due to the large multitude of pharmacological activities of polysaccharides, studies have revealed that polysaccharides from natural plants have multiple effects including antioxidation, anti-inflammation, antitumor, bacteriostatic, and immune regulation (Mei et al., 2020; Wang and Liu, 2020; Ye et al., 2021; Chen et al., 2021b). However, the effect of the SSPs in alleviating gut dysbiosis has not been explored. Especially, How SSPs alter and reshape the gut microbiota remain unknown.

In the present study, we concentrated on the anti-oxidative and anti-inflammatory effects of SSPs in mice and evaluated the intestinal microbiota by 16S rDNA.

## Materials and Methods

### Animals

Male C57BL/6J mice (aged 6–7 weeks, weighing 21–25 g) were purchased from Chengdu Dashuo Laboratory Animal. Animals were housed in groups of six mice with a temperature (22°C ± 3°C), humidity (55% ± 15%), and lighting (12 h light/dark cycle) with *ad libitum* access to food and water. All animals must adapt to conditions for at least 7 days after they arrived. All experimental procedures were approved by the Animal Care and Use Committee of the Sichuan Academy of Agricultural Sciences (Chengdu, China) and were conducted following the academy’s animal experiment guidelines.

### Experimental Design and SSPs Administration

In a 14-day experiment, 18 mice were randomly distributed into three treatment groups with six individuals per group: (1) Control group (CON), gavaged with saline once a day; (2) Low dose SSPs group (LSSP), gavaged with 200 uL SSPs once a day; (3) High dose SSPs group (HSSP), gavaged with 400 uL SSPs once a day. At the end of the experiment, all mice were sacrificed *via* anesthesia using pentobarbital sodium to collect the samples for subsequent determination.

### Enzyme-Linked Immunosorbent Assay

The interleukin-1β (IL-1β), IL-6, IL-10, tumor necrosis factor-α (TNF-α), and interferon-gamma (IFN-γ) in serum and glutathione peroxidase (GSH-Px), superoxide dismutase (SOD), malondialdehyde (MDA), and total antioxidant capacity (T-AOC) contents were determined using spectrophotometric kits according to the manufacturer’s instructions (Nanjing Jiancheng Bioengineering Institute, China).

### Western Blotting

Frozen jejunal samples (approximately 0.1 g) were homogenized using 1 ml RIPA buffer. Following this, ultrasonication was performed to break the cells. The lysates were then centrifuged at 10,000 rcf for 20 min at 4°C. The proteins in the supernatant were diluted with 4× Laemmli sample buffer (Bio-RAD, United States) and denatured in a 98°C metal bath for 10 min. Equal amounts of samples were then subjected to SDS-PAGE, and the abundances of phospho-nuclear factor-E2-related factor 2 (p-Nrf2; Catalog#EP1809Y, Abcam), heme oxygenase-1 (HO-1; Catalog#10701-1-AP, Proteintech), Kelch-like ECH-associated protein 1 (Keap1; Catalog#8047S, Cell Signaling Technology) and GAPDH (Catalog#60004-1-Ig, Proteintech) proteins were assessed by western blot using the indicated antibodies. The expression level of GAPDH was assessed to ensure equal protein sample loading.

### Gut Microbiota Analysis

Samples of the mice’s intestinal contents were collected immediately after sacrifice. The cetyltrimethylammonium bromide/sodium dodecyl sulfate extraction method was employed to obtain the total DNA from the intestinal content. The extracted DNA was subjected to 16S amplification using primers designed to incorporate both the Illumina adapters and a sample barcode sequence, allowing directional sequencing that covers the variable region V4 (primers: 515 F [GTGCCAGCMGCCGCGGTAA] and 806 R [GGACTACHVGGGTWTCTAAT]). Phusion® High-Fidelity PCR Master Mix (New England Biolabs, United States) was used for the PCR reactions.

Sequencing libraries were produced using an Ion Plus Fragment Library Kit 48 rxns (Thermo Scientific, United States) according to the manufacturer’s recommendations. Libraries were sequenced on an Ion S5TM XL platform and 400/600 bp single-end reads were generated. The data were based on sequenced reads and operational taxonomic units (OTUs). UPARSE software (v7.0.1001) was used to carry out the analysis. Sequences that have similarities ≥97% are regarded as the same OTUs. The Silva database was employed to annotate the taxonomic information based on the Mothur algorithm (Xu et al., 2021b).^1^

### Statistical Analysis

All results were analyzed statistically by one-way analysis of variance (ANOVA) tests using IBM SPSS Statistics version 20.0 (IBM, United States) followed by Tukey’s multiple comparison test. The data are expressed in the form of mean ± standard deviation (SD) and *p* < 0.05 was considered to imply statistical difference.

## Results

### Effects of SSPs Administration on the Growth Performance of Mice

**Figure 1:**
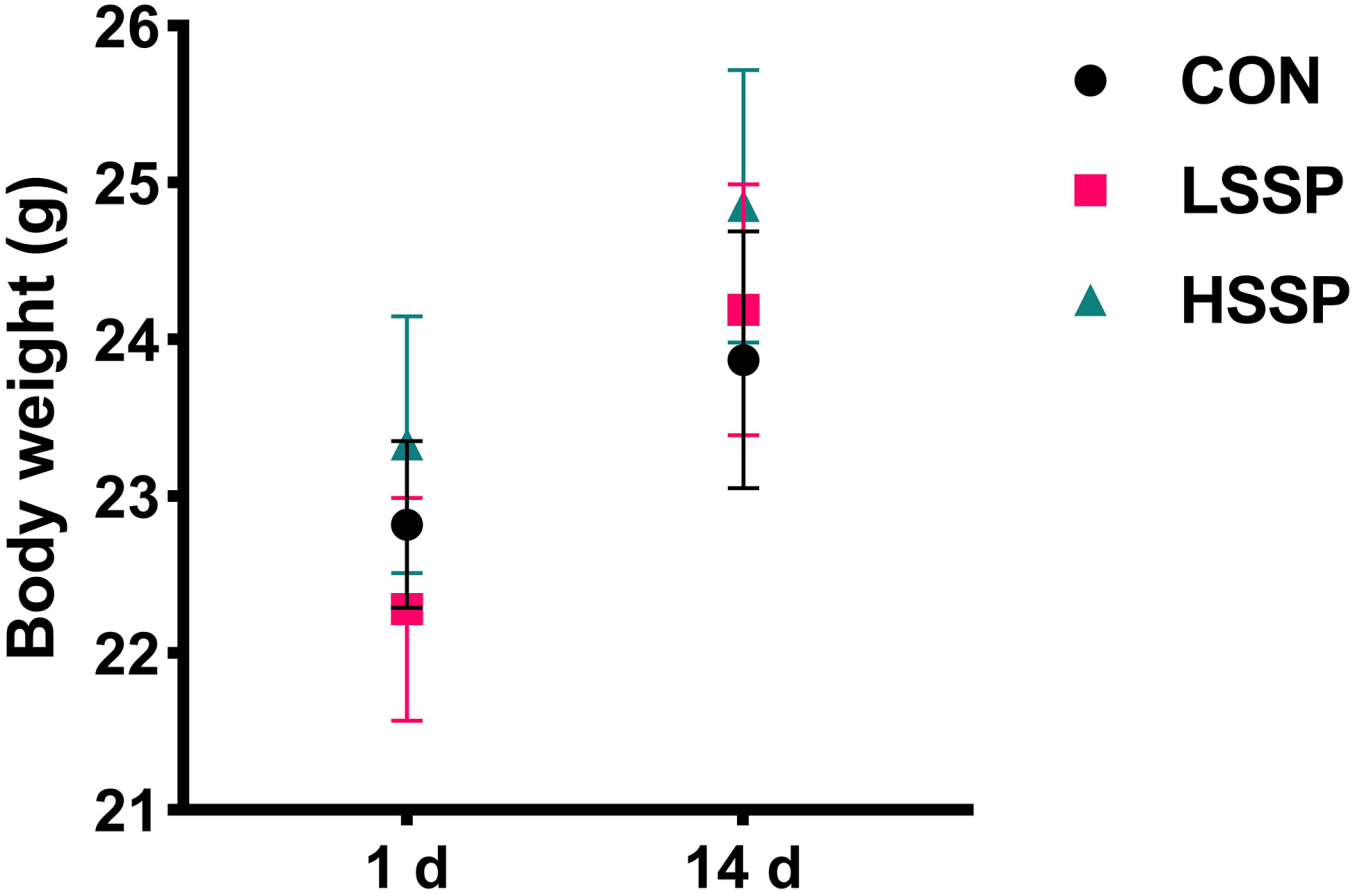
Effect of SSPs on the bodyweight of C57BL/6J mice. CON: control group; LSSP: low dose *Scorias spongiosa* polysaccharide; HSSP: high dose *Scorias spongiosa* polysaccharide.

To determine whether the SSPs administration influences the growth performance of mice, we assessed the body weight (BW) at the beginning and end of the experiment. As shown in Figure 1, there were no significant changes among the three treatment groups (*p* > 0.05), indicating that the growth performance of mice was not affected by SSPs administration.

### Effects of SSPs Administration on Intestinal Inflammation

**Figure 2:**
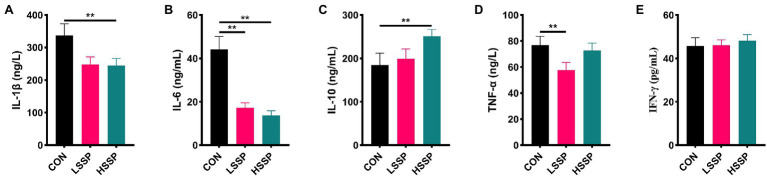
Effect of SSPs on the inflammatory cytokines of C57BL/6J mice. The contents of IL-1β **(A)**, IL-6 **(B)**, IL-10 **(C)**, TNF-α **(D)**, and IFN-γ **(E)** were detected by ELISA. CON: control group; LSSP: low dose *Scorias spongiosa* polysaccharide; HSSP: high dose *Scorias spongiosa* polysaccharide. ^*^Compared with the CON group, ^**^p < 0.01.

To verify the anti-inflammatory capacity of SSPs, some inflammatory cytokines were determined by ELISA. The anti-inflammatory cytokines IL-10 (Figure 2C) increased by HSSPs administration (*p* < 0.01). Moreover, the pro-inflammatory cytokines including IL-1β (Figure 2A), IL-6 (Figure 2B), and TNF-α (Figure 2D) decreased by SSPs in a dose-dependent manner (*p* < 0.01).

### Effects of SSPs Administration on Intestinal Anti-Oxidant Capacity

**Figure 3:**
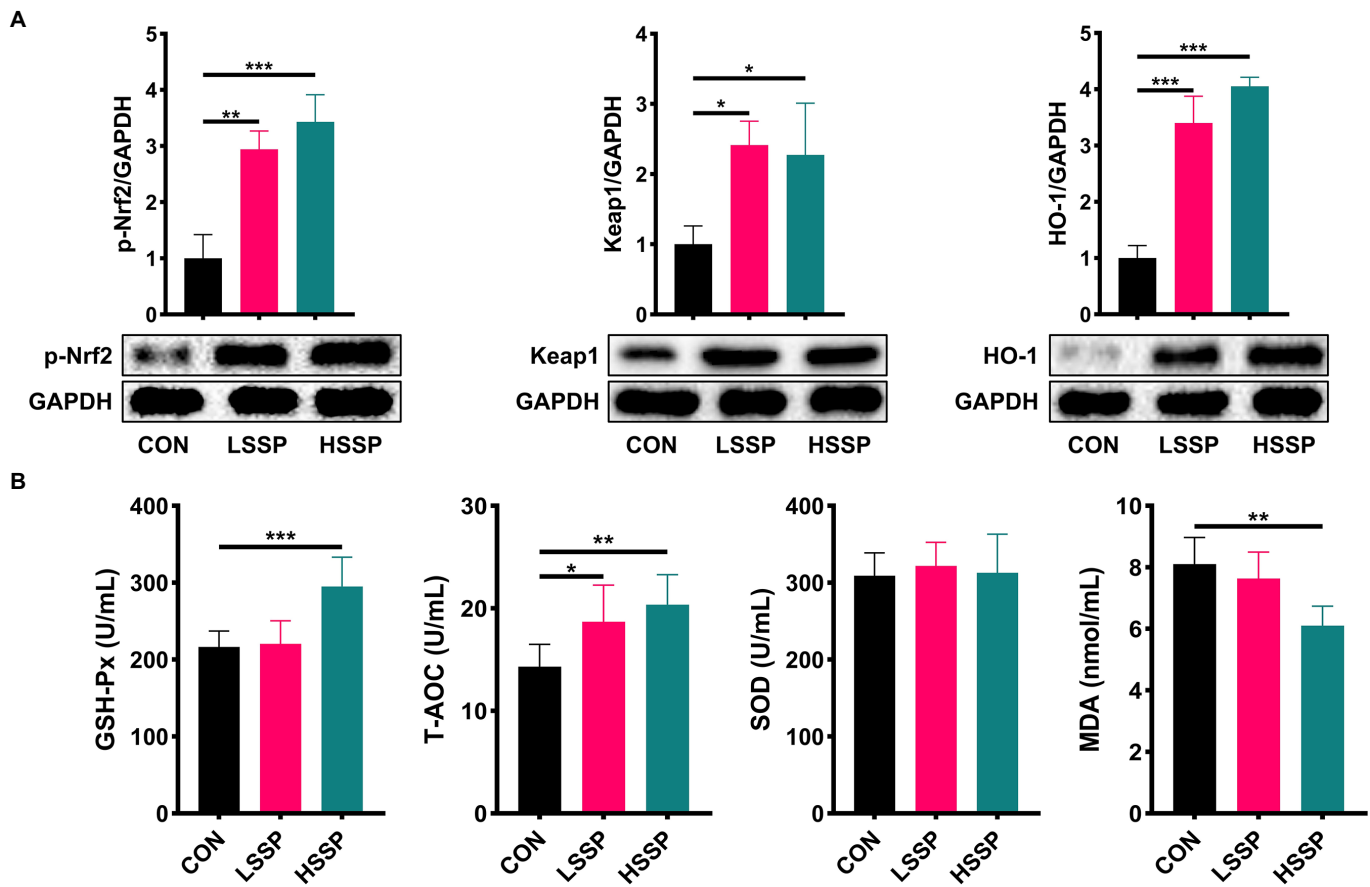
Effect of SSPs on the anti-oxidant capacity of C57BL/6J mice. The protein abundances of p-Nrf2, Keap1 and HO-1 were detected by western blotting **(A)**. The contents of MDA, T-AOC, SOD, and GSH-Px were detected by ELISA **(B)**. CON: control group; LSSP: low dose *Scorias spongiosa* polysaccharide; HSSP: high dose *Scorias spongiosa* polysaccharide. ^*^Compared with the CON group, ^*^p < 0.05, ^**^p < 0.01, and ^***^p < 0.01.

From the results of Figure 3A, it is found that SSPs administration increased (*p* < 0.01) the jejunal protein abundances of Keap1, HO-1, and p-Nrf2 in C57BL/6J mice. Subsequently, we detected the biomarkers of membrane lipid peroxidation and protein oxidative injury. Compared with the control group, SSPs administration decreased the content of MDA and increased the content of GSH-Px and T-AOC.

### Effects of SSPs Administration on Intestinal Microbial Diversity

**Figure 4:**
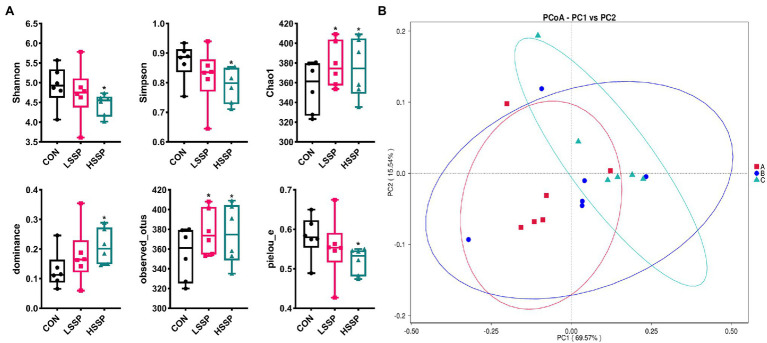
Effect of SSPs on the intestinal bacteria diversity of C57BL/6 J mice. The alpha diversity of intestinal bacteria in C57BL/6 J mice were detected by 16S rDNA **(A)**. The PCoA **(B)** score plots demonstrate complete separation of the jejunal samples among the groups. A (CON): control group; B (LSSP): low dose *Scorias spongiosa* polysaccharide; C (HSSP): high dose *Scorias spongiosa* polysaccharide. ^*^Compared with the CON group, ^*^p < 0.05.

As shown in Figure 4A, SSPs increased (*p* < 0.05) the Chao1 index, dominance index and observed_otus index of bacteria in mice. Meanwhile, HSSPs decreased (*p* < 0.05) the Shannon index, Simpson index and pielou_e index of bacteria in mice. In addition, the PCoA analysis revealed that microbial community was significantly altered by HSSPs administration, with an evident separation (*p* < 0.05) compared with the control group.

### Effects of SSPs Administration on Intestinal Microbiota Composition

**Figure 5:**
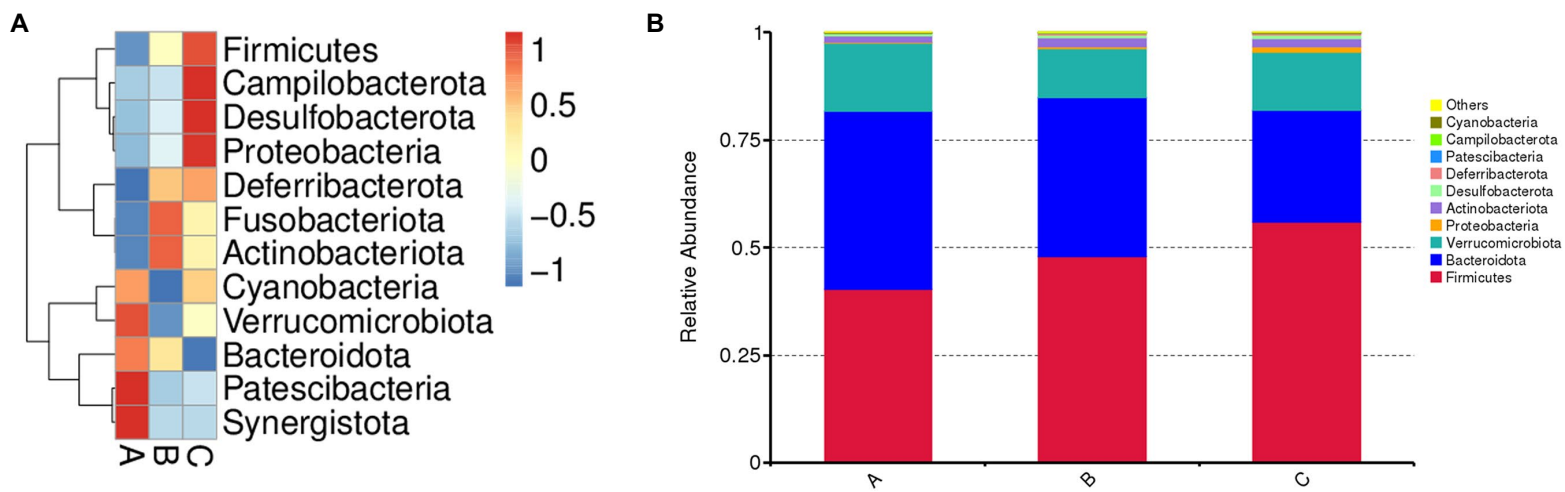
Effect of SSPs on the intestinal bacteria composition of C57BL/6 J mice. Microbial composition of the CON, LSSP, and HSSP groups at the phylum level **(A)**. Relative abundances of microbial composition among three groups at the phylum level **(B)**. A (CON): control group; B (LSSP): low dose *Scorias spongiosa* polysaccharide; C (HSSP): high dose *Scorias spongiosa* polysaccharide.

The bacterial composition was analyzed at different taxonomic levels (Figure 5). At the phylum level, the dominant bacteria were Firmicutes, Bacteroidota, and Verrucomicrobiota, followed by Proteobacteria, Actinobacteria, Desulfobacterota, Deferribacteres, Patescibacteria, Campilobacterota, and Cyanobacteria. SSPs administration increased the abundances of Firmicutes, Campilobacterota, Desulfobacterota, Proteobacteria, Actinobacteria, and Fusobacteria, Bacteroidetes, and Verrucomicrobia, decreased the abundances of Verrucomicrobiota, Bacteroidota, Patescibacteria, and Synergistota.

## Discussion

Edible fungi, which belong to the phylum fungi, can form large fleshy (or colloidal) fruiting bodies or sclerotia tissues and can be used for food or medicine (Guo et al., 2021). Polysaccharides extracted and purified from the fruiting body or mycelium have multiple physiological functions. It is reported that *premna microphylla* turcz leaves polysaccharides (pPMTLs) showed high anti-inflammation activity *via* various pathways including antimicrobial peptides (AMPs) expression pathway, immunodeficiency (IMD) pathway, target of rapamycin (TOR) pathway and intestinal autophagy pathway (Song et al., 2021). Moreover, *Morchella importuna* polysaccharides (MIPs) attenuate CCl_4_-induced hepatic inflammatory injury *via* decreasing pro-inflammatory cytokine production through inhibiting the TLR4/NF-κB signaling pathway (Wen et al., 2019; Xu et al., 2021a). However, the anti-inflammatory activity of the newly found SSPs has not been evaluated. In the present study, the SSPs administration increased the anti-inflammatory cytokines content (IL-10). Contrary to the anti-inflammatory cytokines, IL-1β, IL-6, and TNF-α, as pro-inflammatory cytokines which have been reported in the intestinal inflammation occurrence (Singh et al., 2020; Kim et al., 2021), were dose-dependently decreased by SSPs administration. The results suggested that the anti-inflammation activity of SSPs was relevant to keeping the balance of pro- and anti-inflammatory cytokines.

MDA, as an indirect indicator of the degree of tissue peroxidation, can promote the infiltration of inflammatory cells and even promote the expression of myeloperoxidase (MPO), while SOD, as an antioxidant enzyme, has the opposite effect (Tsikas, 2017; Rosa et al., 2021). GSH-Px is an important enzyme that catalyzes the decomposition of hydrogen peroxide and can specifically catalyze the reduction reaction of GSH to hydrogen peroxide, which plays an important role in protecting cells and tissues from oxidative stress injury (Pu et al., 2022). In our study, the results showed that SSPs administration significantly downregulated the content of MDA and upregulated the content of GSH-Px and T-AOC. To elucidate the molecular mechanisms by which SSPs promoted the anti-oxidative capacity, we investigated the Keap1-Nrf2-ARE signaling pathway-related protein expression.

Oxidative stress refers to that when the body is stimulated, the content of reactive oxygen species (ROS) or reactive nitrogen species (RNS) free radicals exceeds the range that can be cleared by itself, resulting in the imbalance of redox balance and tissue damage, and eventually lead to a series of diseases. In response to oxidative stress, body-self-expression produces a series of endogenous antioxidant factors, including antioxidant molecules and detoxifying enzymes. Among them, the Keap1-Nrf2-ARE signaling pathway plays a crucial role in mediating endogenous antioxidant factors. The results of western blotting indicated that the SSPs administration enhanced the protein level of p-Nrf2, Keap1, and HO-1, implying that SSPs possess a strong anti-oxidant capacity *via* regulating the antioxidant factor production through activating the Keap1-Nrf2-ARE signaling pathway.

The human gastrointestinal (GI) tract is a huge and complex micro-ecosystem, hosting trillions of microorganisms including but not limited to bacteria, fungi, and viruses (Watanabe et al., 2021). The interactions and homeostasis among gut microorganisms, nutrient metabolism and epithelium of the GI tract are critical to host health (Motta et al., 2021). Disrupting the composition of GI microbiota may result in the development and progression of diseases. Numerous studies have demonstrated that the dysbiosis of gut bacteria may interrupt intestinal and systemic immune homeostasis, leading to the development of various kinds of diseases (Baumler and Sperandio, 2016; Sittipo et al., 2018; Chakaroun et al., 2020). As food-derived nutrients could directly interact with gut microbiota and alter the composition, diversity and function of gut bacteria, we found that SSPs-administered mice exhibit more diversity of evenness and richness than those in control mice, as they have higher Chao1 and observed_otu indices. Furthermore, the results of intestinal microbiota composition demonstrated that *Firmicutes* and *Bacteroidota* which is dominated in the gut microbiota were altered by SSPs administration. These species matter as they play a role in the body’s energy-balance mechanism as they affect energy transformation, nutrient absorption, and glucose metabolism. The *Firmicutes*/*Bacteroidetes* ratio is raised in the groups treated with SSPs. These results suggested that SSPs may regulate intestinal diversity and composition to benefit gut health.

## Conclusion

Taken together, our study showed that SSPs are instrumental in intestinal health by enhancing the anti-inflammation and anti-oxidative capacity. Furthermore, the diversity and composition of intestinal microbiota were enriched by SSPs administration. Our findings provided a deep understanding of the benefit of polysaccharides from edible fungi as bioactive materials to intestinal health.

## Data Availability Statement

The datasets presented in this study can be found in online repositories. The names of the repository/repositories and accession number(s) can be found at: NCBI BioProject—PRJNA808190.

## Ethics Statement

The animal study was reviewed and approved by the Animal Care and Use Committee of the Sichuan Provincial People’s Hospital (Chengdu, China).

## Author Contributions

YX, WP, and YW conceived and designed the experiments. ZZ, HF, JT, and YX conducted the experiments. YX and YW wrote the paper. YX, YC, and JZ analyzed the data. All authors contributed to the article and approved the submitted version.

## Funding

This work was financially supported by the China Agriculture Research System of MOF and MARA (CARS-20).

## Conflict of Interest

The authors declare that the research was conducted in the absence of any commercial or financial relationships that could be construed as a potential conflict of interest.

## Publisher’s Note

All claims expressed in this article are solely those of the authors and do not necessarily represent those of their affiliated organizations, or those of the publisher, the editors and the reviewers. Any product that may be evaluated in this article, or claim that may be made by its manufacturer, is not guaranteed or endorsed by the publisher.

## Footnotes

^1^ https://www.arb-silva.de/
